# αPD-L1–γδ T cell conjugates: expanding function beyond direct tumor killing

**DOI:** 10.1093/nsr/nwaf358

**Published:** 2025-09-02

**Authors:** Xindi Wang, Peng Wu

**Affiliations:** Department of Molecular and Cellular Biology, The Scripps Research Institute, USA; Department of Molecular and Cellular Biology, The Scripps Research Institute, USA

Discovered by Brenner about 40 years ago, gamma delta (γδ) T cells are relative newcomers in the field of cancer immunotherapy. Compared to conventional alpha beta (αβ) T cell therapies, γδ T cell-based approaches hold distinct clinical advantages, including major histocompatibility complex (MHC)-independent antigen recognition and low immunogenicity—features that enable repeated allogeneic infusions without eliciting strong immune responses. However, unmodified γδ T cells often exhibit limited tumor-targeting capacity and suboptimal activation, which restrict their therapeutic potential. Enhancing γδ T cell functionality remains a major challenge as current genetic engineering techniques, such as viral transduction and electroporation, are constrained by issues of safety, cost, efficiency and scalability.

As previously reported, the use of chemoenzymatic labeling of cell-surface glycans to construct antibody–cell conjugates represents a highly effective strategy to enhance tumor-specific targeting [1]. Alternatively, metabolic incorporation of unnatural sugars bearing bioorthogonal handles provides a simple and complementary route for antibody–cell conjugation via click chemistry. In a recent study published in *National Science Review*, Chen and coworkers employed this strategy to generate programmed death-ligand 1-specific nanobody (αPD-L1)–γδ T cell conjugates [2]. These engineered cells exhibited enhanced recognition of PD-L1-positive tumor cells and remodeled the tumor microenvironment (TME) through activation of the CCR5/CCL5 chemokine axis.

Specifically, the team employed a fast metabolic glycan labeling (fMGL) technique to label different cell-surface monosaccharides, identifying sialic acids as optimal anchoring sites for tumor-targeting antibodies. Among various azido analogs tested, AMS-ManNAz-P emerged as the most effective unnatural sugar, enabling rapid, high-density labeling at low concentrations (25 µM) or within short incubation time (2 h). Importantly, fMGL labeling minimally affected γδ T cell viability or endogenous function.

**Figure 1.**
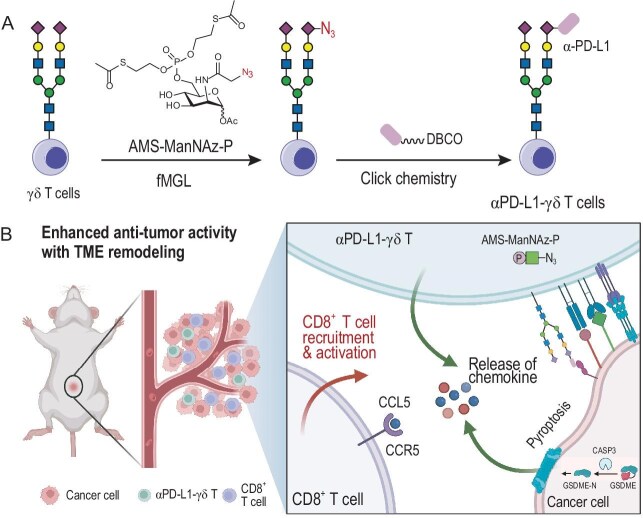
Construction of αPD-L1–γδ T cell conjugates and their tumor-eliminating mechanisms. (A) Using the fast metabolic glycan labeling (fMGL) technique, AMS-ManNAz-P was incorporated into the terminal sialic acids of various cell-surface glycans on γδ T cells. The modified γδ T cells were then conjugated with αPD-L1 via copper-free click chemistry to produce the αPD-L1–γδ T cell conjugates. (B) The targeting antibody αPD-L1 on the conjugates first facilitates γδ T cell binding to PD-L1-expressing cancer cells, which subsequently triggers interactions between TCR γδ and BTN3A1/2A1, co-stimulatory receptors and ligands, as well as death ligands and receptors. This activation leads γδ T cells to release cytotoxic cytokines. In cancer cells, caspase-3 is activated and cleaves gasdermin E (GSDME), initiating pyroptosis. Within the TME, CCL5 was released from the culture of αPD-L1–γδ T cells and tumor cells, thereby recruiting and activating CD8⁺ T cells via the CCR5/CCL5 axis, ultimately remodeling the TME. Adapted from ref. [2]. Figure 1B was created with BioRender.com.

By co-incubating fMGL-labeled γδ T cells with cyclooctyne-functionalized αPD-L1, approximately 6 × 10^7^ αPD-L1 molecules were conjugated per γδ T cell (Fig. 1A). The resulting modification was simple, efficient and durable: αPD-L1 signals persisted for at least 96 h and resisted enzymatic removal by sialidase, likely due to steric protection. These conjugated γδ T cells selectively recognized PD-L1-expressing tumor cells, triggering γδ T cell activation, cytokine release and tumor cell pyroptosis. Notably, the conjugates recruited and activated CD8⁺ T cells through the CCR5/CCL5 axis, with the infiltrating CD8⁺ T cells retaining a central memory phenotype, thereby establishing a ‘hot’ TME that supports durable antitumor immunity (Fig. 1B) [3].

Together, this work identifies optimal sugar molecules for γδ T cell modification, establishing a rapid, robust and minimally disruptive method for antibody conjugation. Beyond preserving γδ T cell function, this approach reduces exhaustion associated with prolonged *in vitro* manipulation, facilitating streamlined cell product preparation. These findings highlight αPD-L1–γδ T cell conjugates as a promising platform for next-generation cancer immunotherapies.
